# Transition from intravenous to enteral ketamine for treatment of nonconvulsive status epilepticus

**DOI:** 10.1186/s40560-017-0248-6

**Published:** 2017-08-08

**Authors:** Michael A. Pizzi, Prasuna Kamireddi, William O. Tatum, Jerry J. Shih, Daniel A. Jackson, William D. Freeman

**Affiliations:** 1 Department of Neurology, Mayo Clinic, 4500 San Pablo Road, Jacksonville, FL 32224 USA; 2 Department of Pharmacy, Mayo Clinic, Jacksonville, FL USA; 3 Present Address: Department of Neurology, University of California, San Diego, CA USA

**Keywords:** Enteral ketamine, Intravenous ketamine, Nonconvulsive status epilepticus, Refractory status epilepticus, Seizures

## Abstract

**Background:**

Nonconvulsive status epilepticus (NCSE) is a diagnosis that is often challenging and one that may progress to refractory NCSE. Ketamine is a noncompetitive *N*-methyl-d-aspartate antagonist that increasingly has been used to treat refractory status epilepticus. Current Neurocritical Care Society guidelines recommend intravenous (IV) ketamine infusion as an alternative treatment for refractory status epilepticus in adults. On the other hand, enteral ketamine use in NCSE has been reported in only 6 cases (1 adult and 5 pediatric) in the literature to date.

**Case presentation:**

A 33-year-old woman with a history of poorly controlled epilepsy presented with generalized tonic-clonic seizures, followed by recurrent focal seizures that evolved into NCSE. This immediately recurred within 24 h of a prior episode of NCSE that was treated with IV ketamine. Considering her previous response, she was started again on an IV ketamine infusion, which successfully terminated NCSE. This time, enteral ketamine was gradually introduced while weaning off the IV formulation. Treatment with enteral ketamine was continued for 6 months and then tapered off. There was no recurrence of NCSE or seizures and no adverse events noted during the course of treatment.

**Conclusion:**

This case supports the use of enteral ketamine as a potential adjunct to IV ketamine in the treatment of NCSE, especially in cases without coma. Introduction of enteral ketamine may reduce seizure recurrence, duration of stay in ICU, and morbidity associated with intubation.

## Background

Nonconvulsive status epilepticus (NCSE) constitutes about 70% of refractory status epilepticus (SE) [1]. Due to varied presentations and dependency on continuous electroencephalography (EEG) for confirmation, diagnosis of NCSE is often delayed, leading to prolonged seizures that can become refractory to standard pharmacotherapy. Current Neurocritical Care Society guidelines and several studies recommend the use of anesthetic agents for refractory SE [2–4]. However, studies have shown poor outcomes with the use of anesthetic agents due to hypotension, respiratory suppression requiring mechanical ventilation, and infections [5–7]. Thus, their use remains controversial in the management of NCSE, particularly in patients presenting with mild confusion or focal clonic jerking [8, 9].

Ketamine may be a better alternative to standard anesthetic agents as it does not cause hypotension or respiratory suppression [10, 11]. Moreover, intravenous (IV) ketamine is known to control 60% of episodes of SE when used as a third- or fourth-line agent [12]. Oral ketamine has been used as an analgesic adjuvant in a variety of chronic pain syndromes [13], but its use in NCSE has been reported in only 6 cases so far, with successful outcomes and no adverse events [14, 15].

We report a case of successful control of NCSE with IV ketamine infusion and gradual transition to enteral ketamine without recurrence or adverse events. This is a potentially useful regimen for refractory NCSE, especially in patients with tenuous blood pressures and those at higher risk from intubation.

## Case presentation

A 33-year-old woman with a 27-year history of poorly controlled focal seizures and focal seizures evolving to bilateral convulsions presented to our clinic. She had multiple prior hospitalizations resulting in the addition, deletion, or dose adjustments of several antiseizure drugs (ASDs). Her ASDs at the time of presentation included phenobarbital 60 mg in the morning and 90 mg at night and levetiracetam 1500 mg, topiramate 150 mg, and lacosamide 200 mg twice daily. She could not tolerate clobazam or valproate (the latter due to elevated liver functions).

Prior to this episode, the patient had 2 hospitalizations in the same month. During the first, she was given propofol and midazolam to control SE, which resulted in hypotension and respiratory failure managed successfully with fluids and mechanical ventilation, respectively. During the second admission, she presented with focal seizures and was found to have NCSE on EEG successfully treated with an IV ketamine infusion of 0.5 mg/kg/h that was tapered off over 2 days. The day after discharge from that hospitalization, she had recurrence of a generalized tonic-clonic seizure at her residence, which lasted approximately 45 s. After termination of her generalized tonic-clonic seizure, she remained confused with development of unilateral facial and eye jerking. She was unresponsive for 10 min until she reached the emergency department (ED).

In the ED, the patient’s mental status was waxing and waning with a Glasgow Coma Scale score of 10. She also had subtle bilateral eye twitching with occasional left gaze deviation and left beating horizontal nystagmus. Eventually, she developed intermittent bilateral upper extremity subtle jerking movements and the Glasgow Coma Scale score improved to 14 with mild confusion.

The patient had no seizure precipitants such as infection, stress, sleep difficulties, or medication noncompliance. Her noncontrasted magnetic resonance imaging and head computed tomography were negative for structural abnormalities. Analysis of cerebrospinal fluid was negative for an infectious etiology, and her paraneoplastic panel, done during a hospitalization within the past month, was negative. Her metabolic profile was negative for any electrolyte disturbances, and her drug screen was negative. ASD levels were within the therapeutic ranges.

**Fig. 1:**
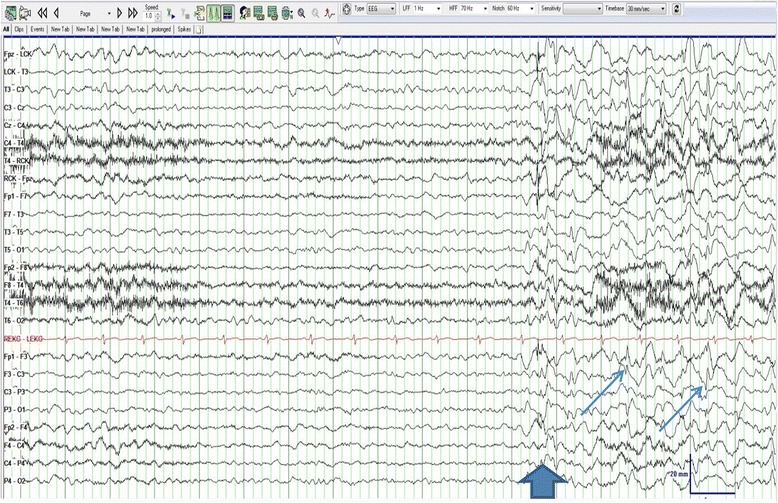
Onset of NCSE (*thick arrow*) on initial EEG after admission. This epoch of the EEG shows onset of NCSE with diffuse slowing of 2- to 2.5-Hz delta and left greater than right hemispheric spikes (*thin arrows*)

The patient’s STAT EEG showed rhythmic 2.0- to 2.5-Hz delta activity with occasional spike-and-wave morphology consistent with electrographic seizure (Fig. 1). The electrographic and clinical findings were consistent with NCSE, and she was admitted to the neurology intensive care unit. In view of the hypotension and respiratory failure associated with prior anesthetic medications and previous good response with ketamine, IV ketamine infusion was started and titrated to 1.25 mg/kg/h. Enteral ketamine was initiated on hospital day 2 (1 day after IV ketamine was started) and increased each day to a final dose of 250 mg twice daily. Enteral ketamine was prepared according to a previously developed protocol by the Mayo Clinic Department of Pharmacy. Ketamine infusion was decreased starting on the same day as initiation of enteral ketamine and subsequently titrated off by hospital day 5. Table 1 depicts the electrographic, clinical, and ketamine administration events from admission to discharge.

**Table 1:** Electrographic and pharmacologic course in the ICU

Day	Continuous EEG	Clinical features	GCS	IV ketamine, mg/kg/h	Oral ketamine, mg BID
1	1. 9 generalized electroclinical and subclinical seizures 2. Generalized slowing mixed delta and theta frequencies	Slightly confused	14	1.25	Not started yet
2	1. Diffuse frontal intermittent rhythmic delta activity 2. Diffuse amplitude suppression that was bihemispheric and occasional localized slowing in the left frontal and right occipital regions 3. No epileptiform activity or nonconvulsive status was noted during this portion of the record.	Alert and oriented	15	Weaned off from 1.25 to 0.75	50
3	1. Diffuse slowing and loss of the posterior-dominant rhythm, monorhythmic frontal delta activity 2. Occasional sharp waves emanating from the right frontal and paracentral region	Alert and oriented	15	0.75	100
4	Same as day 3, with decreased frequency of sharp waves in the frontal region	Alert and oriented	15	Weaned off from 0.75 to 0.5	200
5	Discontinued	Alert and oriented	15	Stopped	250

*BID* twice a day, *EEG* electroencephalography, *GCS* Glasgow Coma Scale score

After termination of NCSE, the patient received a vagal nerve stimulator and was subsequently discharged home with enteral ketamine 250 mg twice daily, phenobarbital 60 mg in the morning and 90 mg at night, levetiracetam 1500 mg and topiramate 175 mg (increased from 150 mg) twice daily, and perampanel 2 mg at night started on day 3 of admission after discontinuation of lacosamide.

For the next 6 months, the patient had no major episodes of SE and did well except for 3 brief hospitalizations for breakthrough seizures. Her seizure frequency decreased more than 50% with the addition oral ketamine and perampanel. No adverse events or dissociative symptoms were noted. She discontinued enteral ketamine after 6 months by tapering its dosage by 50 mg over a 10-week period. As of her 14-month follow-up visit in our epilepsy clinic, she has had no further events after discontinuing oral ketamine.

## Discussion

SE is defined as 5 min or more of continuous clinical or electrographic seizure activity or recurrent seizure activity without returning to baseline between seizures [2]. As the name suggests, NCSE is SE where electrographic seizure activity is seen on EEG without any convulsions [16] and constitutes about 21 to 47% of all SE cases [17, 18].

Diagnosing NCSE has always been a challenge because of its nonspecific presentation, including altered mental status, behavioral abnormalities, focal twitches, automatisms, nystagmus, and even coma, thus leaving a broad differential diagnosis [2]. EEG criteria for diagnosis of NCSE have been outlined [16], but NCSE may present with other rhythmic and periodic patterns that occur as part of the ictal-interictal continuum [2] and do not clearly meet the criteria for NCSE. Other imaging modalities like fluorodeoxyglucose-positron emission tomography [19], diffusion weighted imaging, and fluid attenuation inversion recovery [16] can support the diagnosis of NCSE, but these findings are often delayed.

Our patient’s clinical course was highly suggestive of NCSE due to recurrent clinical seizures and lack of returning to baseline mental status. She presented to the ED with focal seizures and altered mental status following an episode of generalized convulsive seizures at home. This presentation could have been misdiagnosed as a postictal state, but her previous history of NCSE along with an EEG showing rhythmic 2.0- to 2.5-Hz delta meeting the Salzburg criteria [20] led to the diagnosis.

Our patient’s NCSE was refractory as she was already on 4 ASDs at the time of presentation and did not respond to IV diazepam or IV levetiracetam. SE is designated to be refractory when it fails to respond to the acute administration of 2 ASDs [21]. Several investigators have described the mechanism for refractory seizures to be attributed to internalization of inhibitory γ-aminobutyric acid (GABA)-A receptors and increased expression of excitatory noncompetitive *N*-methyl-d-aspartate (NMDA) and α-amino-3-hydroxy-5-methyl-4-isoxazolepropionic acid (AMPA) receptors on the postsynaptic neuronal membranes [22]. Hence, drugs that act on GABA receptors may lose their efficacy, while drugs that inhibit NMDA (ketamine) or AMPA (perampanel) receptors may have greater efficacy in terminating SE or NCSE lasting more than 30 min. An animal study supports this mechanism by showing ketamine to be more effective in prolonged seizures lasting more than an hour, rather than administered after only 15 min of induced SE [23].

Current guidelines and several studies support the use of anesthetic agents for refractory SE [24]. There is concern over their adverse events on respiratory suppression requiring intubation and severe hypotension. A recent prospective study [25] states that therapeutic coma in SE increases hospital length of stay and related costs. Also, there are observational studies which provide clear evidence of increased mortality and poor outcomes in patients with SE treated with IV anesthetic agents [5–9, 26].

Considering our patient’s mental status (Glasgow Coma Scale score 14), previous refractory NCSE that responded well to IV ketamine, and severe hypotension with prior use of propofol, she was immediately started on IV ketamine infusion in the neuro-ICU. Adding a ketamine infusion to her home ASD regimen allowed for control of her refractory seizures while avoiding over-sedation and subsequent intubation. Furthermore, this treatment regimen did not cause systemic hypotension.

**Fig. 2:**
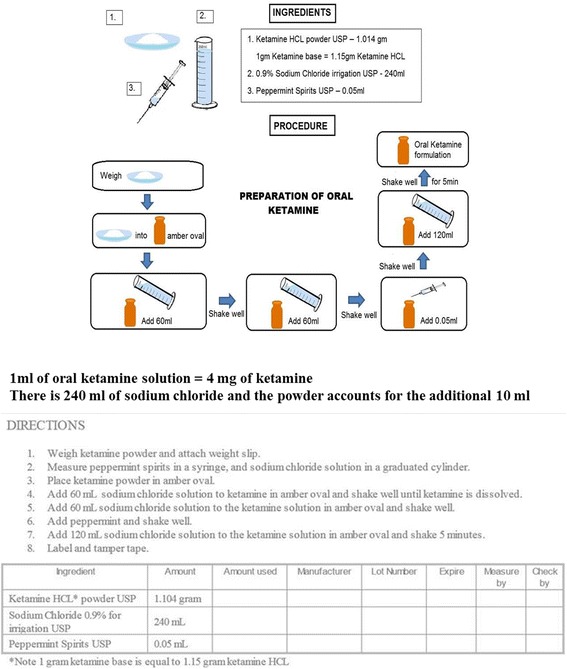
Extemporaneous preparation of enteral ketamine in our pharmacy

IV ketamine has been in use for a long time as an alternative drug for refractory SE, but usage of oral ketamine has been reported in only 6 patients, 1 adult and 5 pediatric patients [14, 15]. There is no commercially available oral preparation, so it was extemporaneously prepared by the hospital pharmacy (Fig. 2). Furthermore, no specific dosage recommendations are available with proven efficacy or safety profile, and published case reports served as a guide to the current regimen.

In the published pediatric cases, oral ketamine was started as a last resort for refractory NCSE. The enteral preparation (50 mg/ml) was given orally at a dose of 1.5 mg/kg/day in 2 divided doses. Enteral ketamine was administered for 5 days in addition to the maintenance ASD treatment and then stopped without weaning. Resolution of NCSE occurred in 24 to 48 h of starting enteral ketamine, and no adverse events were reported [14]. In the adult case, IV ketamine was first given as a bolus of 1.5 mg/kg followed by continuous infusion with gradual dose titration from 0.05 to 0.4 mg/kg/h. She was then switched to enteral ketamine 50 mg twice daily to avoid intubation due to her “Do Not Resuscitate” code status. This patient’s clinical SE and EEG improved and remained stable after a total dosage of 2000 mg of ketamine, which was then withdrawn later in the course of treatment [15]. Based on these published cases of enteral ketamine, we started our patient on enteral ketamine 50 mg twice daily (1.25 mg/kg/day in 2 divided doses), followed by increased titration based on EEG findings.

We initiated an IV ketamine infusion with titration to 1.25 mg/kg/h. Once our patient’s EEG showed resolution of electrographic seizures for 24 h, enteral ketamine 50 mg twice daily was introduced while we began weaning her off the IV ketamine infusion (Table 1). We tapered from 1.25 to 0.75 to 0.5 mg/kg/h, and eventually, it was discontinued. We simultaneously increased the enteral ketamine dosage from 50 to 100 to 200 to 250 mg twice daily, respectively. Enteral ketamine 250 mg twice daily was continued along with her other ASDs for 6 months and then tapered off over a 10-week titration schedule.

The onset of action for IV ketamine is 1 to 5 min and for enteral ketamine 15 to 30 min [27]. Moreover, enteral ketamine undergoes first pass metabolism, which decreases bioavailability and is metabolized to norketamine. This metabolite is much weaker than ketamine and is associated with fewer adverse events [13]. Therefore, IV ketamine can be used to terminate NCSE due to its rapid action and 100% bioavailability and then be transitioned to enteral ketamine with a safer profile as a maintenance therapy to prevent seizure recurrence. Several adverse events, including increased intracranial pressure, dissociative symptoms, and neurotoxicity, have been described in the literature [28], but there were no adverse events seen with any of the previously published patients treated with oral ketamine nor in our patient in spite of continuing treatment for 6 months. She also had better seizure control after discharge from the hospital, but this could be due to a combined effect of enteral ketamine, AMPA antagonist perampanel, and vagus nerve stimulation (VNS). The efficacy of perampanel for the treatment of SE has yielded mixed results in published case reports, with a small series demonstrating EEG improvements seen in 24 to 48 h after starting perampanel for SE [29, 30]. In a review of VNS for refractory SE (RSE), 76% of generalized RSE and 25% of focal RSE resolved after placement of a VNS [31].

## Conclusion

ASD medication management of refractory NCSE is debatable and should be tailored based on the condition of the patient at the time of presentation. Ketamine could be a better choice in refractory NCSE or subtle focal SE than other anesthetic agents, and its early initiation in the course of management may be considered with additional validation from a larger population. Enteral ketamine, though not currently indicated for NCSE, was beneficial as a maintenance therapy in our patient after controlling electrographic seizures with IV ketamine. This case and successful transitional regimen requires further support from prospective, controlled studies.

## Abbreviations

AMPA: α-Amino-3-hydroxy-5-methyl-4-isoxazolepropionic acid
ASDs: Antiseizure drugs
EEG: Electroencephalography
GABA: γ-Aminobutyric acid
IV: Intravenous
NCSE: Nonconvulsive status epilepticus
NMDA: *N*-methyl-d-aspartate
RSE: Refractory status epilepticus
VNS: Vagus nerve stimulator
